# Updated Surveillance Metrics and History of the COVID-19 Pandemic (2020-2023) in East Asia and the Pacific Region: Longitudinal Trend Analysis

**DOI:** 10.2196/53214

**Published:** 2025-02-21

**Authors:** Alexander L Lundberg, Alan G Soetikno, Scott A Wu, Egon Ozer, Sarah B Welch, Yingxuan Liu, Claudia Hawkins, Maryann Mason, Robert Murphy, Robert J Havey, Charles B Moss, Chad J Achenbach, Lori Ann Post

**Affiliations:** 1 Buehler Center for Health Policy and Economics Robert J Havey, MD Institute for Global Health Northwestern University Chicago, IL United States; 2 Department of Emergency Medicine Feinberg School of Medicine Northwestern University Chicago, IL United States; 3 Feinberg School of Medicine Northwestern University Chicago, IL United States; 4 Department of Medicine, Division of Infectious Diseases Feinberg School of Medicine Northwestern University Chicago, IL United States; 5 Center for Pathogen Genomics and Microbial Evolution Robert J. Havey, MD Institute for Global Health Northwestern University Chicago, IL United States; 6 Center for Global Communicable and Emerging Infectious Diseases Robert J Havey, MD Institute for Global Health Northwestern University Chicago, IL United States; 7 Robert J Havey, MD Institute for Global Health Northwestern University Chicago, IL United States; 8 Department of Medicine, General Internal Medicine and Geriatrics Feinberg School of Medicine Northwestern University Chicago, IL United States; 9 Institute of Food and Agricultural Sciences University of Florida Gainesville, FL United States

**Keywords:** SARS-CoV-2, COVID-19, East Asia, Pacific, American Samoa, Australia, Brunei Darussalam, Cambodia, China, Fiji, French Polynesia, Guam, Hong Kong, Indonesia, Japan, Kiribati, People’s Democratic Republic of Korea, Republic of Korea, Lao People’s Democratic Republic, Macao, Malaysia, Marshall Islands, Federated States of Micronesia, Mongolia, Myanmar, Nauru, New Caledonia, New Zealand, Northern Mariana Islands, Palau, Papua New Guinea, Philippines, Samoa, Singapore, Solomon Islands, Thailand, Timor-Leste, Tonga, Tuvalu, Vanuatu, Vietnam, pandemic, surveillance, public health, speed, acceleration, deceleration, jerk, dynamic panel, generalized method of moments, Arellano-Bond, 7-day lag

## Abstract

**Background:**

This study updates the COVID-19 pandemic surveillance in East Asia and the Pacific region that we first conducted in 2020 with 2 additional years of data for the region.

**Objective:**

First, we aimed to measure whether there was an expansion or contraction of the pandemic in East Asia and the Pacific region when the World Health Organization (WHO) declared the end of the COVID-19 *public health emergency of international concern* on May 5, 2023*.* Second, we used dynamic and genomic surveillance methods to describe the dynamic history of the pandemic in the region and situate the window of the WHO declaration within the broader history. Finally, we aimed to provide historical context for the course of the pandemic in East Asia and the Pacific region.

**Methods:**

In addition to updates of traditional surveillance data and dynamic panel estimates from the original study, this study used data on sequenced SARS-CoV-2 variants from the Global Initiative on Sharing All Influenza Data to identify the appearance and duration of variants of concern. We used Nextclade nomenclature to collect clade designations from sequences and Pangolin nomenclature for lineage designations of SARS-CoV-2. Finally, we conducted a 1-sided *t* test to determine whether the regional weekly speed was greater than an outbreak threshold of 10. We ran the test iteratively with 6 months of data across the sample period.

**Results:**

Several countries in East Asia and the Pacific region had COVID-19 transmission rates above an outbreak threshold at the point of the WHO declaration (Brunei, New Zealand, Australia, and South Korea). However, the regional transmission rate had remained below the outbreak threshold for 4 months. In the rolling 6-month window *t* test for regional outbreak status, the final *P* value ≤.10 implies a rejection of the null hypothesis (at the α=.10 level) that the region as a whole was not in an outbreak for the period from November 5, 2022, to May 5, 2023. From January 2022 onward, nearly every sequenced SARS-CoV-2 specimen in the region was identified as the Omicron variant.

**Conclusions:**

While COVID-19 continued to circulate in East Asia and the Pacific region, transmission rates had fallen below outbreak status by the time of the WHO declaration. Compared to other global regions, East Asia and the Pacific region had the latest outbreaks driven by the Omicron variant. COVID-19 appears to be endemic in the region, no longer reaching the threshold for a pandemic definition. However, the late outbreaks raise uncertainty about whether the pandemic was truly over in the region at the time of the WHO declaration.

## Introduction

### Background

COVID-19, the disease caused by SARS-CoV-2, was first detected in Wuhan, China, in the fall of 2019 [1-5]. Our research team conducted an analysis of the pandemic in East Asia and the Pacific region one year into the pandemic [6]. This study provides 2 additional years of updated surveillance and analysis for the region.

We adopted the World Bank’s definition of East Asia and the Pacific region, which is based on economic development and geographical proximity, encompassing American Samoa, Australia, Brunei Darussalam, Cambodia, China, Fiji, French Polynesia, Guam, Hong Kong, Indonesia, Japan, Kiribati, People’s Democratic Republic of Korea, Republic of Korea, Lao People’s Democratic Republic, Macao, Malaysia, Marshall Islands, Federated States of Micronesia, Mongolia, Myanmar, Nauru, New Caledonia, New Zealand, Northern Mariana Islands, Palau, Papua New Guinea, the Philippines, Samoa, Singapore, Solomon Islands, Thailand, Timor-Leste, Tonga, Tuvalu, Vanuatu, and Vietnam [7]. North Korea neither collects nor reports COVID-19 caseloads and deaths.

The World Health Organization (WHO) and Director-General Ghebreyesus declared the end of *COVID-19 as a public health emergency of international concern* on May 5, 2023 [8-10], based on the recommendation of the COVID-19 Emergency Committee [10]. We compared the progression of the pandemic before and after this declaration.

### Empirical Definition of Pandemic Versus Epidemic Versus Outbreak Versus Endemic

Epidemiological terms, such as pandemic, epidemic, outbreak, and endemic, are used to describe the occurrence and spread of disease [11,12]. The distinctions between these terms lie in their scope, geographic extent, and severity. An epidemic refers to a sudden increase in the number of disease cases in a specific population or region. If the epidemic spreads across several countries or continents, it becomes a pandemic. An outbreak, on the other hand, describes a sudden increase in a concentrated setting, usually involving a more limited geographic area than an epidemic. Endemic refers to the constant presence of a disease in a particular geographic region or population, with no sudden increases in case volume [13,14]. Field epidemiology defines these terms based on transmission metrics and geographical distribution and uses transmission metrics and geographical distribution to differentiate the nuances of these terms. Although public health surveillance data may not capture all cases, they are the best proxy for monitoring and tracking disease spread within a population in near real time. Surveillance is crucial for the timely response to health threats [15].

### Traditional Surveillance Versus Enhanced Surveillance

Public health surveillance is the “ongoing, systematic collection, analysis, and interpretation of health-related data essential to planning and evaluation of public health practice” [16]. Not only does surveillance explain the burden of death and disease transmission but also it generates research questions and guides investigators on topics that require further investigation [17-31]. Surveillance allows us to compare the burden of disease between geographical regions and to understand which regions are most impacted. The impact can be measured through per capita rates of how many people contract a disease, how many die, and affiliated costs.

However, traditional surveillance carries several limitations that this study has addressed. Traditional surveillance provides a snapshot of past events [17-31], meaning it is static and only reflects historical data. In the middle of a burgeoning pandemic, policy makers and public health practitioners also need to understand what is about to happen. Is an outbreak increasing? Will growth switch from linear to exponential? Are more people dying from that particular condition in one place than another? To inform health policy and practice, knowledge of what is about to happen is often more valuable than knowledge of what did happen. To that end, we have developed enhanced surveillance metrics that reflect the dynamics of a pandemic and inform imminent growth—most importantly, where along the epidemiological outbreak curve a particular region is situated. In addition, we included dynamic metrics about the speed of the pandemic at the national, regional, and global level. We measured how acceleration of speed one week compared to the prior week, as well as how novel infections in a prior week predicted new cases the following week. We can think of the latter measure as the echoing forward of cases. These metrics were tested and validated in prior research [6,32-42].

For the purpose of this study, standard surveillance metrics explain what has already happened in East Asia and the Pacific region, while enhanced surveillance metrics provide insights into future trends or the current position of a country along an epidemiological curve. We used both types of metrics to analyze the possible end to the pandemic in East Asia and the Pacific region.

### Objectives

This study has 3 objectives. First, we aimed to measure whether there was an expansion or contraction in the pandemic in East Asia and the Pacific region at the time the WHO declared the end of the *COVID-19 pandemic as a public health emergency of international concern* on May 5, 2023 [43-51]. At both the region and country level, we used advanced surveillance and analytical techniques to describe the status of the pandemic in a 2-week window around the WHO declaration. From a public health perspective, we needed to know whether the rate of new COVID-19 cases was increasing, decreasing, or stable from week to week, and if any changes in the transmission rate indicated an acceleration or deceleration of the pandemic. Statistical insignificance is significant—it can signal the epidemiological *end* to the pandemic if the rate of new cases is 0 (or very low) and stable, meaning the number of new cases is neither accelerating nor decelerating.

Second, we used dynamic and genomic surveillance methods to describe the history of the pandemic in the region and situate the time window around the WHO declaration within the broader history. We included the ratio of COVID-19 deaths to the number of transmissions as a proxy for the mortality risk from infection at the population level. We also included a historical record of genomic surveillance from sequenced viral specimens to identify the appearance and spread of variants of concern in the region.

Third, we aimed to provide historical context for the course of the pandemic in East Asia and the Pacific region. We have addressed several questions. How did countries respond to the pandemic? How did the region fare in terms of disease burden? And what social, economic, and political factors shaped the course of COVID-19 in the region? This context can provide important lessons for disease prevention and mitigation in future pandemics.

## Methods

### Overview

We conducted trend analyses with longitudinal COVID-19 data from Our World in Data (OWID) [52]. This study provides updates of traditional surveillance data and dynamic panel estimates from the original work by Post et al [6,40,41,53-55]. For East Asia and the Pacific region, the data comprised an unbalanced panel of 36 countries and territories, running from August 14, 2020, to May 12, 2023. Because several countries around the world switched from daily to weekly reports at various points in 2023, we used a cubic spline to interpolate daily new cases and deaths if any country had 4 consecutive periods of nonzero new cases interspersed by 6 days of 0 new cases [56-58].

To identify the appearance and duration of variants of concern, we also used data on sequenced SARS-CoV-2 variants from the Global Initiative on Sharing All Influenza Data (GISAID), which is an effective and trusted web-based resource for sharing genetic, clinical, and epidemiological COVID-19 data [59-62]. We used Nextclade nomenclature [63] to collect clade designations from sequences and Pangolin nomenclature for lineage designations of SARS-CoV-2 [64,65]. Metadata for the study period were collected on June 22, 2023. To avoid low frequency or potentially erroneous samples, the dataset was further filtered to exclude months with fewer than 100 available samples, variant groups with fewer than 5 samples in a month, and variant groups representing <0.5% of the total samples in a month. The final dataset consisted of 184,386 total samples available on GISAID [59-62].

Traditional surveillance metrics include the *speed* of transmission or the rate of new COVID-19 cases per 100,000 population. Novel metrics include *acceleration*, *jerk*, and *1- and 7-day persistence* estimates. *Acceleration* is the change in speed from one time unit to the next. Acceleration can measure whether transmission rates are rising, falling, or stable. *Jerk* is the change in acceleration from one time unit to the next. The term *jerk* is adopted from physics nomenclature, as a large jerk can signal explosive growth in transmissions. The *1- and 7-day persistence* measures provide the impact of the 1- and 7-day lag of speed on current speed. For another interpretation, these estimates capture how COVID-19 cases echo forward to cases either 1 or 7 days later. They are coefficient estimates from an Arellano-Bond dynamic panel data model [66]:



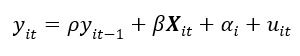



in which the dependent variable is speed, the independent variables include weekend and recent week indicators, *α_i_* is a country fixed effect, and *u_it_* is the idiosyncratic error term. Advantages of the Arellano-Bond estimates include a correction for time-invariant omitted variables and the ability to assess both the predictive ability of the model and validity of its specification [41].

We analyzed the potential *statistical end* to the pandemic with a 1-sided *t* test for whether the mean of speed was equal to or greater than the outbreak threshold of 10. We ran the test on a rolling 6-month window over weekly speed for the region, and we plotted the *P* values from the test over time. The test can assess whether the region as a whole was experiencing an outbreak over a defined time window, during which the speed may sometimes exceed the outbreak threshold and at other times remain below it. All statistical analyses were conducted in R (version 4.2.1; R Foundation for Statistical Computing) with the *plm* package (version 2.6-2) [53,54].

### Ethical Considerations

All data used in this study are publicly available and contain no identifiable, private information. Therefore, the study is deemed exempt research with human subjects as defined by the US Government Code of Federal Regulations 45CFR46 and so was not submitted the Northwestern University Institutional Review Board. However, the authors note that anonymized COVID-19 data surveillance systems can generate local and global ethical questions beyond the scope of this study [67].

## Results

Tables 1-6 present traditional (ie, static) and novel (ie, dynamic) COVID-19 surveillance metrics with OWID transmission and death data for East Asia and the Pacific region. These results focus on the 2-week period around the time when the WHO declared COVID-19 was no longer a public health emergency of international concern. The week before the declaration was April 28, 2023, and the week after was May 5, 2023.

**Table 1:** Arellano-Bond dynamic panel data estimates of the number of daily COVID-19 infections reported by country in East Asia and the Pacific region from April 28 to May 12, 2023a,b.

Variables	Values	*P* values^c^
1-day lag coefficient	0.171	.46
7-day lag coefficient	0.877	<.001
Shift parameter, week of April 28	−0.488	<.001
Shift parameter, week of May 5	0.491	<.001
Weekend effect	−1.215	.28

^a^Wald test: χ^2^_6_=730,275, *P*≤.001.

^b^Sargan: χ^2^_540_=24, *P*=.99.

^c^Contains estimates from the model in equation (1).

**Figure 1:**
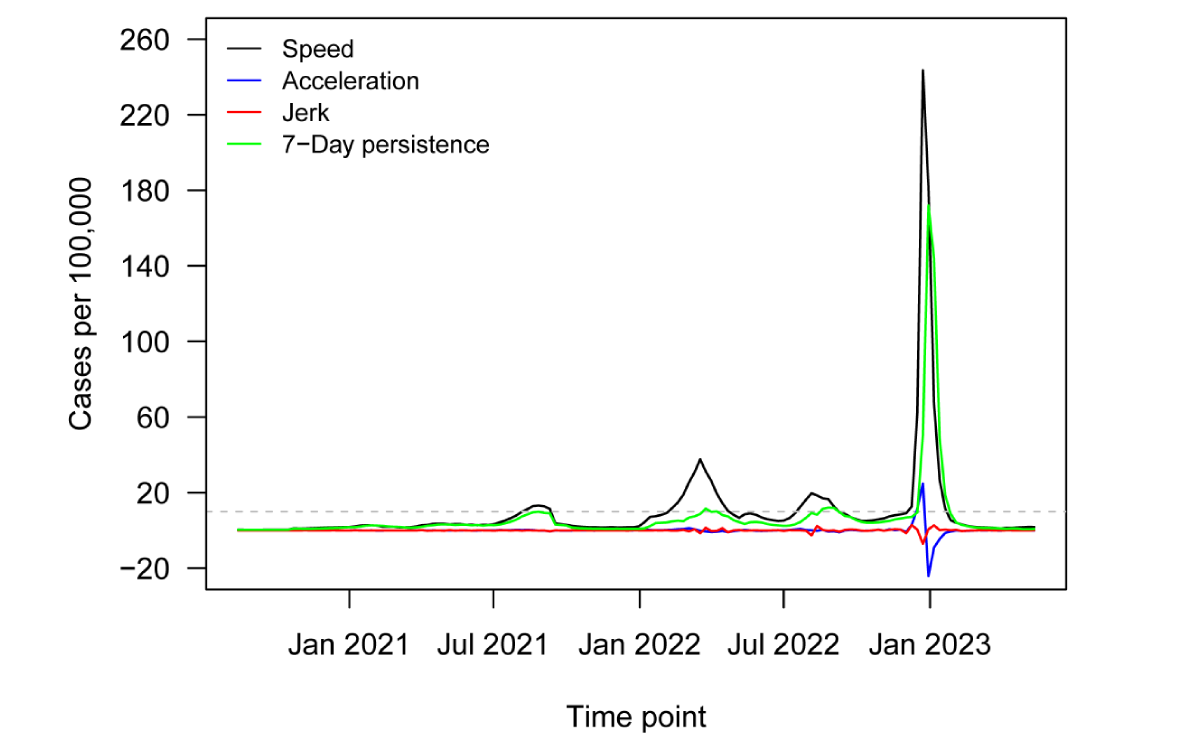
Novel surveillance metrics (speed, acceleration, jerk, and 7-day persistence) for COVID-19 infections in East Asia and the Pacific region from August 2020 to May 2023.

**Figure 2:**
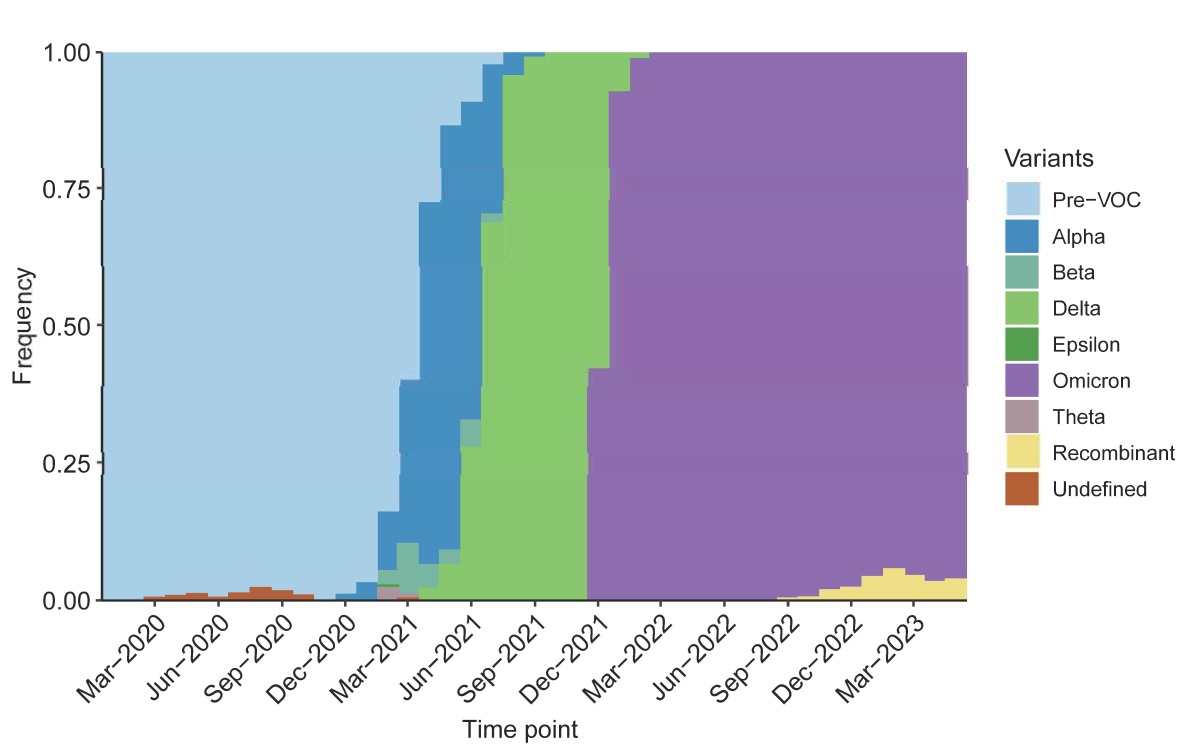
Variants of concern (VOCs) as a proportion of all sequenced SARS-CoV-2 specimens from March 2020 to May 2023 in East Asia and the Pacific region (n=1,234,493).

**Figure 3:**
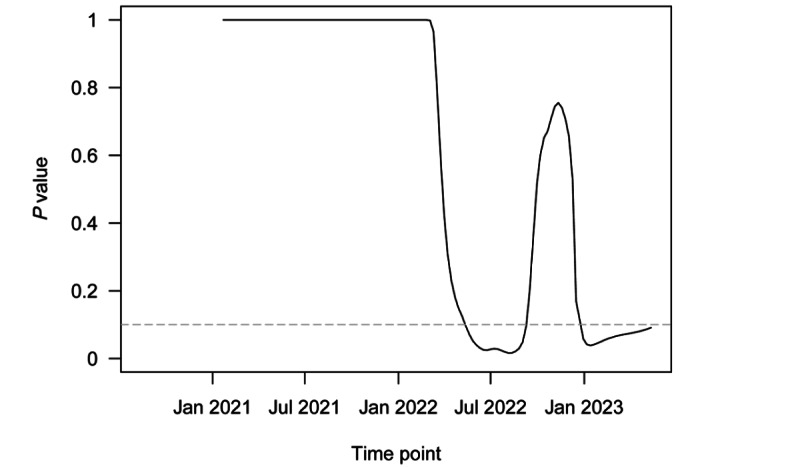
*P* values from t tests of weekly COVID-19 transmissions per 100,000 population equal to 10 over a rolling 6-month window in East Asia and the Pacific region.

**Figure 4:**
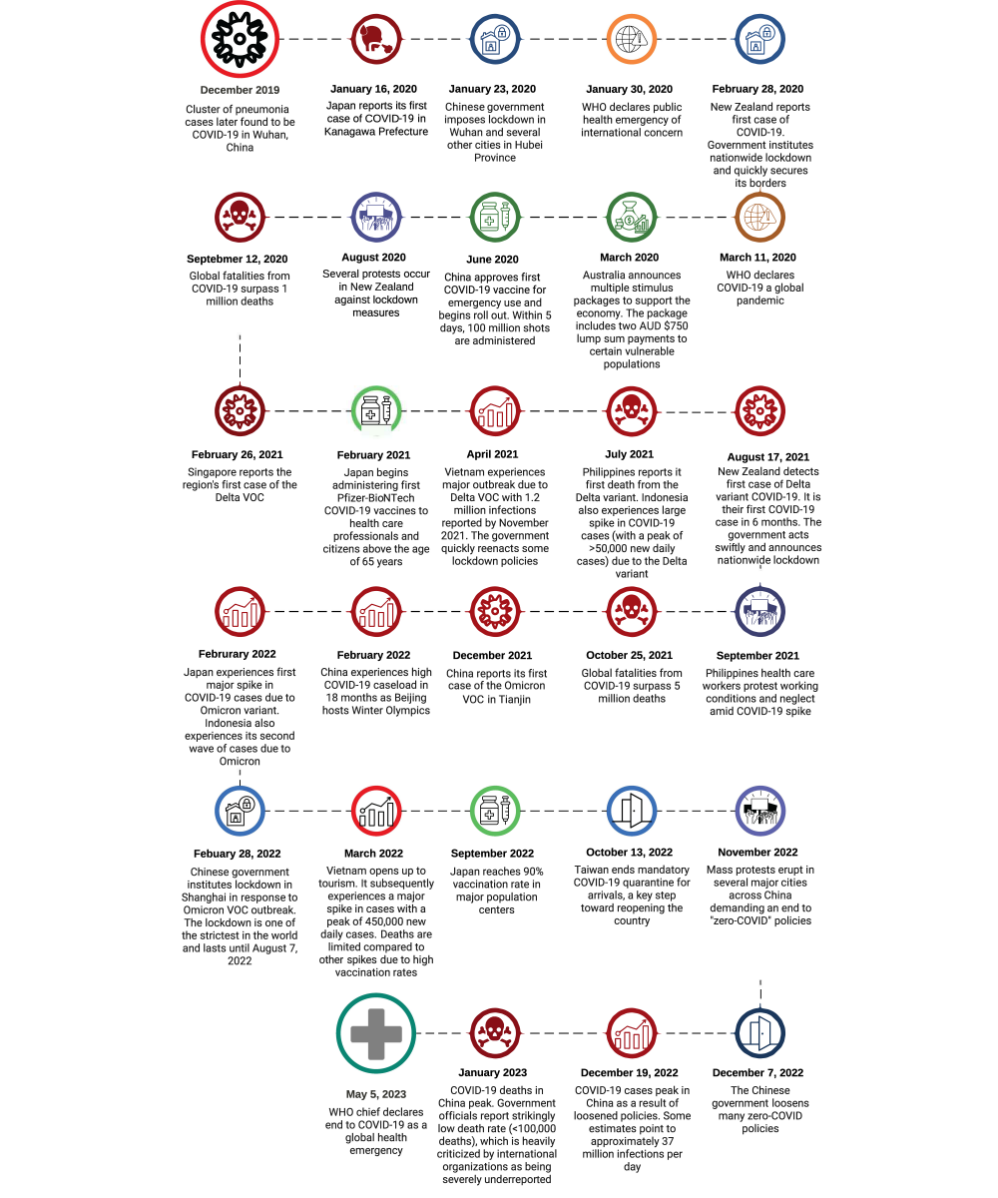
Timeline of the COVID-19 pandemic in East Asia and the Pacific region. VOC: variant of concern; WHO: World Health Organization.

Figure 1 plots the novel surveillance metrics over time, from the start of the pandemic up to the WHO declaration in May 2023. Figure 2 uses GISAID data on sequenced SARS-CoV-2 variants to present the proportion of variants of concern returned among specimens over a similar period. Figure 3 returns to regional transmission data to present the results of a rolling 6-month window *t* test for whether the region was in a state of outbreak over each period. Finally, Figure 4 presents a qualitative timeline of key events in the COVID-19 pandemic in East Asia and the Pacific region.

Table 1 presents the dynamic panel estimates for the most recent time window. The Wald test for the regression was significant (*P*<.001), and the Sargan test failed to reject the validity of the overidentification restrictions (*P*=.99). The 1-day lag coefficient was not statistically significant (*P*=.46), but the 7-day lag coefficient was (*P*<.001), suggesting a cluster effect in which cases on a given day impact cases 7 days later. The shift parameter for the week of April 28 was negative (−0.488), while the more recent shift parameter for the week of May 5 was positive (0.491). The roughly equal magnitudes suggest the echo-forward effect of cases remained roughly stable from the start to the end of the 2-week period.

Standard surveillance metrics for the weeks of April 28 and May 5, 2023, are provided in Tables 2 and 3. Although most countries in the region had a small number of new COVID-19 cases, several countries had high rates of transmission. For the week of April 28, Brunei had by far the highest rate of new COVID-19 cases at 1431 per 100,000 population. The next highest rate was 204 in New Zealand, followed by 115 in Australia and 36 in South Korea. These rates exceeded the outbreak threshold according to the Centers for Disease Control and Prevention [32-42,68]. Specifically, *low* transmission is defined as no more than 10 cases per 100,000 people per week, *moderate* transmission ranges from 10 to 50 cases per 100,000 people per week, and *substantial* transmission is between 50 to 100 cases per 100,000 people per week [68,69]. Thus, Brunei, New Zealand, and Australia were in greater-than-substantial outbreaks, while South Korea was in a moderate outbreak.

Outbreak status for countries in the region looked roughly the same for the week of May 5. Brunei had somewhat lower transmission rate than in the prior week, while Australia had a higher rate. The other exception is Guam, who entered an outbreak with a speed of 61 (Table 3). The speed in Guam was 17 for the week of April 21. The dip to zero in the intermediate week (Table 2) most likely represents a disruption in data reports, but it is also important to note that transmission speed can be more variable in small island territories. For the region as a whole, COVID-19 may have still met the pandemic definition at the time of the WHO declaration, with several countries experiencing sizeable outbreaks. However, transmission rates were low for the rest of the region, suggesting a transition from pandemic to endemic.

**Table 2:** Static COVID-19 surveillance metrics for East Asia and Pacific countries in the week of April 28, 2023.

Country	New COVID-19 cases, n	Cumulative COVID-19 cases, n	7-day moving average of new cases	Infection rate per 100,000 individuals	New weekly deaths, n	Cumulative deaths, n	7-day moving average of deaths	Death rate per 100,000 individuals	Conditional death rate
Australia	4301	11,272,355	4273.57	115.01	0	20,613	18	0.52	0
Brunei	918	292,644	721.29	1431.20	0	158	0.14	0.30	0
Cambodia	0	138,733	0.14	0	0	3056	0	0	0.02
China	462	99,250,200	511.14	0.03	7	120,984	5.86	0	0
Fiji	0	68,921	0	0	0	883	0	0	0.01
French Polynesia	6	78,518	9	0	0	649	0	0	0.01
Guam	0	51,240	8.71	0	0	413	0	0	0.01
Indonesia	2122	6,784,170	1871.14	0.77	20	161,404	20.86	0.01	0.02
Japan	7343	33,766,957	11,059.29	5.92	18	74,614	21	0.01	0
Laos	4	218,085	1.29	0.05	0	671	0	0	0
Malaysia	0	5,071,840	709	0	0	37,020	1.29	0	0.01
Mongolia	19	1,008,038	9.43	0	0	2136	0	0	0
Myanmar	208	635,660	140.57	0.38	0	19,492	0.14	0	0.03
New Caledonia	0	80,058	0	0	0	314	0	0	0
New Zealand	1509	2,261,126	1423.57	203.73	0	2762	3.71	0.51	0
Northern Mariana Islands	3	13,849	1.86	0	0	41	0	0	0
Palau	0	6000	0	5.53	0	9	0	0	0
Papua New Guinea	1	46,850	1.14	0.01	0	670	0	0	0.01
Philippines	1190	4,097,525	932.71	1.03	0	66,444	0	0	0.02
Singapore	3201	2,391,770	3316.71	0	0	1722	0	0	0
South Korea	18,752	31,251,203	15,477.57	36.19	6	34,518	6.71	0.01	0
Thailand	242	4,732,301	252.29	2.36	0	33,957	1.43	0.01	0.01
Timor	2	23,431	0.43	0.15	0	138	0	0	0.01
Vietnam	2233	11,567,728	1836.14	2.27	0	43,195	1	0	0

**Table 3:** Static COVID-19 surveillance metrics for East Asia and Pacific countries in the week of May 5, 2023.

Country	New COVID-19 cases, n	Cumulative COVID-19 cases, n	7-day moving average of new cases	Infection rate per 100,000 individuals	New weekly deaths, n	Cumulative deaths, n	7-day moving average of deaths	Death rate per 100,000 individuals	Conditional death rate
Australia	5076	11,303,671	4643.29	135.74	0	20,751	19.71	0.43	0
Brunei	816	299,505	927.86	1271.72	0	160	0.29	0.54	0
Cambodia	0	138,736	0.43	0	0	3056	0	0	0.02
China	572	99,254,488	612.57	0.04	12	121,048	9.14	0	0
Fiji	0	68,921	0	0	0	883	0	0	0.01
French Polynesia	3	78,545	3.57	0	0	649	0	0	0.01
Guam	105	51,345	15	61.12	0	413	0	0	0.01
Indonesia	1471	6,795,221	1578.71	0.53	22	161,574	24.29	0.01	0.02
Japan	833	33,803,572	5230.71	0.67	25	74,694	11.43	0.02	0
Laos	4	218,096	1.57	0.05	0	671	0	0	0
Malaysia	0	5,079,436	1085.14	0	0	37,028	1.14	0	0.01
Mongolia	49	1,008,265	36.43	0	0	2136	0	0	0
Myanmar	157	636,967	186.71	0.29	0	19,493	0.14	0	0.03
New Caledonia	0	80,058	0	0	0	314	0	0	0
New Zealand	1535	2,272,229	1564.43	207.28	0	2792	4.29	0.89	0
Northern Mariana Islands	4	13,872	3.57	0	0	41	0	0	0
Palau	1	6000	0.14	0	0	9	0	0	0
Papua New Guinea	0	46,864	2	0	0	670	0	0	0.01
Philippines	1940	4,108,914	1627	1.68	0	66,453	1.29	0	0.02
Singapore	3386	2,414,394	3277	0	0	1722	0	0	0
South Korea	19,989	31,371,347	17,163.43	38.58	8	34,591	10.43	0.02	0
Thailand	320	4,734,000	275.43	3.12	0	33,967	1.43	0.03	0.01
Timor	1	23,435	0.57	0.07	0	138	0	0	0.01
Vietnam	2823	11,585,390	2523.14	2.88	0	43,200	0.71	0	0

A comparison of Tables 2 and 3 demonstrates little to no change before and after the WHO declared an end to COVID-19 as a public health emergency. China, Japan, and South Korea had the most cumulative cases of COVID-19 transmissions, but these ranks are a function of population size. Thus, a better measure is the number of COVID-19 cases and deaths per 100,000 population. Moreover, death is often a better proxy for the state of an outbreak than transmissions because deaths are less likely to be undercounted [70]. Undercounting may be due to poor public health infrastructure, increased use of home antigen testing, or a dearth in polymerase chain reaction testing or other resources. China, Japan, and South Korea reported <.01 deaths per population. When we control for risk of death given the number of COVID-19 transmissions, we find that Myanmar had the highest conditional death rate, at 0.03 deaths per 100,000 population, while Cambodia, Indonesia, and the Philippines each reported a rate of 0.02.

**Table 4:** Novel COVID-19 surveillance metrics for East Asia and Pacific countries for the week of April 28, 2023.

Country	Speed	Acceleration	Jerk	7-day persistence effect on speed
Australia	114.28	0.31	−0.02	39.51
Brunei	1124.51	111.65	−4.97	134.57
Cambodia	0	0	0	0
China	0.04	0	0	0.01
Fiji	0	−0.05	−0.05	0.02
French Polynesia	3.26	0	0	0.51
Guam	5.07	−2.49	0.75	2.52
Indonesia	0.68	0	−0.03	0.15
Japan	8.92	−0.51	−0.90	2.88
Laos	0.02	0	−0.01	0.01
Malaysia	2.09	0	0	0.71
Mongolia	0.16	0	0	0.04
Myanmar	0.26	0.03	0	0.04
New Caledonia	0	0	0	0.02
New Zealand	192.18	2.01	1.31	72.76
Northern Mariana Islands	2.88	0	0	2.54
Palau	0.79	0.79	0.79	0
Papua New Guinea	0.01	0	0	0
Philippines	0.81	0.05	0.01	0.16
Singapore	59.34	0	0	24.83
South Korea	29.87	1.37	−0.17	8.68
Thailand	2.46	−0.01	−0.02	0.71
Timor	0.03	0.02	0.02	0.01
Vietnam	1.87	−0.11	0.12	0.85

Tables 4 and 5 present enhanced dynamic surveillance metrics for the weeks before and after May 5. For most countries, speed was low and stable, which implied values of acceleration and jerk close to 0. Four countries were in a state of outbreak: Australia, Brunei, New Zealand, and South Korea.

From the first to the second week, acceleration was positive for Australia but negative for Brunei, which means the outbreak was worsening in Australia but improving in Brunei. The near-zero value of jerk for Australia suggests an inflection point of the outbreak, which may have been on the verge of reduction. Because acceleration was negative for Brunei, the negative value of jerk suggests a continued, but slowed, downward trend in the transmission rate. However, the 7-day persistence effect remained high for both countries.

The outbreaks in New Zealand and South Korea remained stable, with near-zero acceleration and jerk, although the 7-day persistence effect showed a slight increase. Because 4 out of the 36 countries and territories were experiencing outbreaks, the distinction between pandemic and endemic for the region remains unclear. On the basis of trends in other global regions, these outbreaks are likely to be the last outbreaks of this magnitude for these 4 countries; however, the scale of these outbreaks means that transmissions could spread across borders, potentially leading to new outbreaks in neighboring countries.

Finally, we note that the figures in Tables 4 and 5 are not calculated as day-over-day averages across the week, as in Tables 2 and 3. Thus, the magnitudes of speed may not exactly match those presented in Tables 2 and 3.

**Table 5:** Novel COVID-19 surveillance metrics for East Asia and Pacific countries for the week of May 5, 2023.

Country	Speed	Acceleration	Jerk	7-day persistence effect on speed
Australia	124.16	2.96	0.61	44.39
Brunei	1446.57	−22.78	−25.05	436.81
Cambodia	0	0	0	0
China	0.04	0	0	0.01
Fiji	0	0	0	0
French Polynesia	1.26	0	0	1.27
Guam	8.73	8.73	8.73	1.97
Indonesia	0.57	−0.03	0.05	0.26
Japan	4.22	−0.75	1.17	3.47
Laos	0.02	0	0.01	0.01
Malaysia	3.20	0	0	0.81
Mongolia	0.95	0	0	0.06
Myanmar	0.34	−0.01	−0.01	0.10
New Caledonia	0	0	0	0
New Zealand	211.21	0.51	−1.12	74.65
Northern Mariana Islands	6.63	0	0	1.12
Palau	0	−0.79	−0.79	0.31
Papua New Guinea	0.02	0	0	0
Philippines	1.41	0.09	0.02	0.31
Singapore	57.34	0	0	23.05
South Korea	33.12	0.34	0.14	11.60
Thailand	2.69	0.11	0.03	0.96
Timor	0.04	−0.01	−0.01	0.01
Vietnam	2.57	0.09	−0.10	0.73

**Table 6:** East Asia and Pacific countries with the highest 7-day persistence estimate in the week of May 5, 2023.

Country	7-day persistence (May 5, 2023)
Brunei	436.81
New Zealand	74.65
Australia	44.39
Singapore	23.05
South Korea	11.60

Table 6 compares the 7-day persistence effect on speed for the top 5 countries for the week of May 5. Unsurprisingly, persistence was highest for Brunei, which had the largest outbreak at the time. Interestingly, Singapore appears on the list despite not having reached outbreak status. The persistence suggests that an outbreak could have been imminent for Singapore. Alternatively, the persistence could signify a strong, continued presence of COVID-19 as endemic in the area.

Figure 1 plots regional speed, acceleration, jerk, and 7-day persistence metrics from August 14, 2020, to May 12, 2023. The dashed gray line denotes the informal Centers for Disease Control and Prevention outbreak threshold of speed equal to 10. The region saw 4 outbreaks over the period, but the first and third were small, barely eclipsing a speed of 10. The first outbreak happened in August 2021. The second happened in February 2022, ultimately reaching a peak speed of 38. A third minor outbreak occurred in July 2022. The final outbreak began in December 2022, reaching a peak speed of 243 at the end of the year. This outbreak was one of the largest in the world over the course of the pandemic. However, since the end of January 2023, the region has not been in a state of outbreak.

Figure 2 plots variant groups as a proportion of all viral specimens collected and sequenced in the region (and made available through GISAID) each month. The first outbreak occurred shortly after the appearance of the Delta variant. Each subsequent outbreak occurred after the dominant Omicron variant arrived. East Asia and the Pacific region, like much of the rest of the world, saw a surge in cases amid the heightened transmissibility of Omicron [71]. In contrast, the large outbreaks driven by Omicron did not occur until the end of 2022. This delay was achieved by the effective zero–COVID-19 policy in China, which was finally abandoned at that point. The outbreak hit in force shortly afterward.

Another potential indication of the end to the pandemic is the continued dominance of the Omicron variant. While the region saw a mixture of the ancestral, Alpha, Beta, and Delta variants before the arrival of Omicron in November of 2021, viral sequences have almost exclusively been identified as Omicron and its subvariants ever since.

Figure 3 plots *P* values from a series of 1-sided *t* tests of whether speed for the region was equal to or greater than the threshold outbreak of 10. These tests were conducted on a rolling 6-month window of weekly regional speed. The test can assess whether the region was in an outbreak over a period in which speed may sometimes eclipse an outbreak threshold and other times remain below the threshold. Statistical insignificance indicates no evidence against the null hypothesis of below-outbreak regional speed over a period. For example, at the α=.10 level, the final *P* value ≤.10 in the figure implies a rejection of the null hypothesis that the region as a whole was not in an outbreak for the period from November 5, 2022, to May 5, 2023.

In Figure 3, the dashed gray line denotes the least restrictive conventional significance level threshold of α=.10. The test first rejected the null in favor of the alternative for the 6-month period ending in early May of 2021. The test lost significance again in mid-September, before regaining significance at the very end of 2022. This second period of rejection was driven by the large Omicron outbreak around the time. More recently, the test was on the verge of insignificance at the time of WHO declaration. Although the test failed to reject the null at the α=.10 level, it did reject at the α=.05 level. Compared to other global regions, East Asia and the Pacific region are the latest to potentially transition from the pandemic to endemic COVID-19 status.

Within the historical context of enhanced surveillance metrics, the region may be at the end stage of the pandemic. Speed has not been this low for this long since the start of the pandemic. However, we do note that several countries in the region remained in substantial outbreaks at the time of the WHO declaration. This reality brings some uncertainty to the ostensible end to the pandemic in the region.

Figure 4 provides a timeline of the onset of COVID-19 in East Asia and the Pacific region as well as vaccination programs and major events that shaped the progression of the pandemic in the region. Most notably, the zero–COVID-19 policy in China effectively contained the disease in the country and therefore to an extent in the region. Upon the abandonment of the policy, a large wave of cases driven by Omicron finally hit the country, much later than in other parts of the world.

## Discussion

### Principal Findings

COVID-19 has affected all countries in East Asia and the Pacific region. As of March 16, 2023, the WHO had reported a total of 213,883,614 COVID-19 cases and 622,018 deaths in East Asia and the Pacific region [72]. At the same point, the WHO reported 760,360,956 confirmed cases of COVID-19, including 6,873,477 deaths worldwide [72].

The results indicate a potential COVID-19 transition from pandemic to endemic at the time the WHO removed the designation of COVID-19 as a public health emergency of international concern on May 5, 2023. Regional speed had remained below the outbreak threshold of 10 for several months ahead of the WHO declaration. The rolling *t* test of speed, equal to the outbreak threshold, suggests that the region was in an outbreak over the six-month period starting at the end of 2022 when the Omicron variant drove a large increase in cases. However, the result was only significant at the α=.10 level, not the 0.05 level. However, several countries in the region had transmission rates consistent with a substantial outbreak in the 2 weeks around the WHO declaration. These outbreaks make an apparent end to the pandemic in the region unclear at the time.

As another indicator, nearly all sequenced SARS-CoV-2 specimens in the region were identified as the Omicron variant from early 2022 onward. This transition is consistent with a more clearly defined end to the pandemic in other global regions [73-75]. The relative delay for East Asia and the Pacific region may be attributable to a late Omicron outbreak in the region when China relaxed its stringent and effective zero–COVID-19 policy [76].

### Policies Implemented to Control and Mitigate the Transmission of COVID-19

Many countries in the region experienced multiple waves of COVID-19 infections, with some waves more severe than others. During the initial outbreaks, countries in East Asia and the Pacific region were quick to respond with policies that were highly effective at curbing transmission rates and limiting mortality. Compared to other regions of the world, Asian countries experienced significantly lower mortality early in the pandemic (2.7 deaths per million in Asian countries vs 197 deaths per million in European countries) [77]. This disparity has been attributed to several factors.

First, countries in the region were able to quickly formulate effective strategies that were guided by their prior experiences with other respiratory pandemics, such as the 2003 outbreak of SARS-CoV-1 and the 2015 outbreak of Middle East respiratory syndrome [78-80].

Second, cultural differences between Eastern and Western societies may have allowed countries to more easily adopt face masking and social distancing policies [77]. Where Western countries such as the United States pride themselves on individual freedoms, many Asian countries have cultures that value collective societal well-being [81].

Third, the region as a whole tended to implement strict containment policies, which typically included mask mandates, social distancing, mass testing requirements, contact tracing, quarantine protocols, border lockdowns, and eventually mass vaccination programs to control the virus.

China initially implemented a strict zero–COVID-19 policy with the aim to keep cases as close to zero as possible. At times, entire cities, such as Shanghai, Chengdu, and Wuhan, entered citywide lockdown to prevent the spread of the virus [82-84]. Amid these lockdowns, economic activities were halted, public transportation was suspended, and residents were restricted to their homes. Meanwhile, individuals who tested positive for COVID-19 were taken to large isolation facilities to undergo quarantine [82,85].

New Zealand and Taiwan also initially implemented strict COVID-19 policies including mask mandates, tight border control, contact tracing, and tightly targeted quarantines for local outbreaks. These strategies were highly effective in containing the virus during major outbreaks for both countries [86,87]. However, both eventually shifted away from these strategies with the emergence of more contagious variants such as Delta and Omicron. Instead, they focused more on achieving herd immunity through widespread vaccination campaigns and protecting populations [86,88].

Vietnam, having learned lessons from the SARS outbreak in 2003, began preparing for COVID-19 weeks before the first cases were reported in their country. Knowing that their health care resources were limited, Vietnam’s strategic goal was to keep COVID-19 cases as low as possible. They began stockpiling personal protective equipment and created local medical response teams to address outbreaks. When Vietnam detected its first cases in the community, it acted decisively and swiftly by locking down areas and performing meticulous contact tracing [89,90]. Vietnam never relied on widespread testing to curb infections [89].

The Philippines and Indonesia were COVID-19 hotspots throughout the pandemic due to limited public health responses by their governments. Both countries were late to institute COVID-19 containment measures, such as contact tracing, testing, and isolation, leading to high rates of infections and deaths relative to other countries in the region [91-94]. In the Philippines, the country’s limited health care infrastructure left it ill-prepared for the pandemic, leading to an exponential growth in cases during the first wave [94,95].

Many Pacific Island countries were quick to implement strategies to prevent COVID-19 infections. Given their geographical isolation, these countries went almost completely free of COVID-19 cases during the initial wave of infections through the use of strict border closures and restrictions on international travel [96-98]. For example, the Solomon Islands reported its first COVID-19 case in October 2020 in a student who was returning from the Philippines [99]. However, it was not until 2022 that the Solomon Islands faced its first widespread community outbreak [100]. These strict border closures, while effective, came with a natural economic cost as many island nations rely heavily on tourism, which virtually evaporated during the pandemic [97].

Subsequent waves of infection were primarily driven by local easing of COVID-19 precautions and the emergence of new and more contagious variants such as Delta and Omicron. For example, Japan has experienced several waves of COVID-19 infections, with the largest wave occurring in mid-2022 due to the Omicron variant [101]. China, on the other hand, has reported fewer waves of COVID-19 infections due to its strict containment measures, although it is not clear how accurate their data are given their lack of consistent reporting. However, when China eased their zero–COVID-19 policy in December 2022, it subsequently experienced an intense surge in cases and related deaths [76]. Other countries in Asia, such as Vietnam and Taiwan, have been relatively successful in controlling the virus and have experienced fewer waves of infection.

While countries enjoyed relative success early on in controlling disease, they had to grapple with the economic and social consequences of their restrictive containment policies. These outbreaks and their associated governmental responses led to substantial sociopolitical turmoil throughout the region. Protests were widespread and focused on the government’s mishandling of economic recovery and overly restrictive COVID-19 policies. Thailand and Malaysia saw protests break out in 2021 due to the government’s perceived lackluster pandemic response [102,103]. The Philippines saw protesters calling for an end to quarantine rules, which led to President Duterte to issue a “shoot to kill” order against the protesters [104,105]. In China, antigovernment protests broke out throughout the country in late 2022 calling for an end to China’s zero–COVID-19 policy, which used strict and draconian city-wide lockdowns to curb the spread of the virus. In response, China eased their policy in December 2022 and subsequently experienced an intense surge in cases and deaths. The WHO criticized China for underrepresenting the true number of deaths, with one senior official stating that China was using too narrow of a definition to count deaths [106,107]. New Zealand saw protesters converge on Parliament to oppose COVID-19 vaccines mandates [108].

In terms of the economy, East Asia and the Pacific region experienced a 0.2% decline in gross domestic product (GDP) in 2020 driven by containment measures that limited economic activity. However, as vaccines became more prevalent and countries began easing restrictions, economies rebounded with a GDP growth of 5.8% in 2021 [109].

To combat the economic ramifications of their containment strategies, governments in the region focused on reopening their economies and using large fiscal packages to stimulate economic recovery. These fiscal packages typically included direct cash support for affected workers, tax relief for individuals and corporations, support for their strained health care systems, and economic investment in specific economic sectors and public works. In 2020, the Chinese government launched several fiscal measures estimated to be worth approximately 4.9 trillion RMB (4.7% of GDP; US $672.3 billion). Key aspects of these measures included tax relief, investment in epidemic prevention and control, and medical equipment [110]. In Vietnam, the government introduced a fiscal support package worth 291.7 trillion VND (3.6% of GDP; US $11.6 billion) with measures that included deferment of value-added, personal income, and corporate income taxes [110]. The Australian government approved a substantial fiscal stimulus package worth AUS $312 billion (15.75% of GDP; US $196 billion) to be used through the financial year 2025, which included an AUS $20 billion (US $12.5 billion) health response package to secure vaccines and strengthen the existing health care system [110]. Singapore announced an SGD $92 billion (US $67.9 billion) fiscal package that included a cash payout to all Singaporeans [110]. In 2020, Japan approved multiple stimulus packages, including the Emergency Economic Package against COVID-19 (worth ¥117.1 trillion; US $750.5 billion) and the Comprehensive Economic Measures to Secure People’s Lives and Livelihoods toward Relief Hope package (worth ¥73.6 trillion; US $471.7 billion). These measures were intended to protect businesses and support wages while also focusing on building a more resilient future economy [110].

### Vaccination Efforts

Efforts to create a vaccine started immediately. SinoVac, a Chinese pharmaceutical company, began development of an inactivated COVID-19 vaccine called CoronaVac as early as January 2020. By fall 2020, SinoVac launched phase 3 trials in several countries, including Brazil, Indonesia, the Philippines, and Turkey [111]. The large phase 3 trial in Brazil, which used a 2-dose series administered 14 days apart, demonstrated an efficacy of 51% against symptomatic COVID-19 infections and 100% against severe infections [112]. By early 2021, many countries in East Asia and the Pacific region, as well as in South America and Eastern Europe, began mass vaccination programs using CoronaVac [113-118]. By April 2021, SinoVac had announced that it had production capacity to produce 2 billion doses per year [111]. On June 1, 2021, the WHO validated the vaccine for emergency use. Other countries, such as Taiwan, Japan, and Korea, purchased millions of vaccines from western countries, including the AstraZeneca, Pfizer-BioNTech, and Moderna vaccines in early 2021 [119-122].

With many countries now having reached herd immunity through a combination of several available vaccines, there has been widespread easing of border restrictions, as seen in Japan, China, Taiwan, Singapore, Australia, and New Zealand [123-127].

### Conclusions

Concerns about potential resurgences of the virus remain valid. As long as COVID-19 continues to spread and mutate, the possibility of new variants emerging remains. These variants could potentially be more transmissible, be resistant to vaccines, or cause more severe illness. This underlines the importance of continued vigilance, vaccination efforts, and global cooperation to control the spread of the virus [39].

### Limitations

COVID-19 data became less frequently reported worldwide by the time the WHO declared an end to the pandemic as a public health emergency of international concern [128]. In addition, more people began using at-home tests as the pandemic evolved [129], and experts believe the Chinese government has underreported COVID-19 deaths [130]. Because the enhanced surveillance metrics of speed, acceleration, jerk, and 7-day persistence are based on rates and not total counts, statistical bias caused by countries dropping in or out of the sample is mitigated. However, if a nonincluded country is unrepresentative of the region in terms of disease burden, its omission can still influence historical data comparisons. Viral specimen tests for variants of concern in GISAID depend on testing and sequencing capacity, which varied by country across the region.

## Abbreviations

GDP: gross domestic product
GISAID: Global Initiative on Sharing All Influenza Data
OWID: Our World in Data
VOC: variant of concern
WHO: World Health Organization
